# Illicit substance exposure in pregnancy and infant mortality risk: a nationwide Taiwan study

**DOI:** 10.1093/ijnp/pyaf046

**Published:** 2025-07-09

**Authors:** Chun Lin, Mu-Hong Chen, Wei-Szu Lin, Shiow-Ing Wu, Yuan-Chun Liao, Yu-Hsuan Lin, Ching-Heng Lin

**Affiliations:** Kunming Prevention and Control Center, Taipei City Hospital, Taipei, Taiwan; Department of Psychiatry, Taipei Veterans General Hospital, Taipei, Taiwan; Department of Psychiatry, College of Medicine, National Yang Ming Chiao Tung University, Taipei, Taiwan; Department of Medical Research, Taichung Veterans General Hospital, Taichung, Taiwan; Institute of Population Health Sciences, National Health Research Institutes, Zhunan, Miaoli County, Taiwan; Division of Controlled Drugs, Taiwan Food and Drug Administration, Taipei, Taiwan; Institute of Population Health Sciences, National Health Research Institutes, Zhunan, Miaoli County, Taiwan; Department of Psychiatry, National Taiwan University Hospital, Taipei, Taiwan; Department of Psychiatry, College of Medicine, National Taiwan University, Taipei, Taiwan; Department of Biomedical Sciences and Engineering, National Central University, Taoyuan, Taiwan; Department of Medical Research, Taichung Veterans General Hospital, Taichung, Taiwan; Department of Public Health, College of Medicine, Fu Jen Catholic University, New Taipei City, Taiwan; Department of Industrial Engineering and Enterprise Information, Tunghai University, Taichung, Taiwan; Institute of Public Health and Community Medicine Research Center, National Yang Ming Chiao Tung University, Taipei, Taiwan; Department of Epidemiology and Public Health, UCL, London, United Kingdom

**Keywords:** substance use, illicit drug use, methamphetamine, polysubstance, infant mortality

## Abstract

**Background:**

To investigate the association between prenatal illicit substance exposure and infant mortality, addressing the unclear links between specific and multiple substances and increased mortality.

**Methods:**

This 16-year retrospective cohort study used Taiwan’s National Health Insurance Research Database, the Taiwan Maternal and Child Health dataset, and the Integrated Illegal Drug Database, including 1 937 301 pregnant women who delivered from 2004 to 2019. Among them, 11 477 used illicit drugs during pregnancy, with a matched control group of 45 908 non-users based on maternal age, income, and childbirth year. Of the drug users, 26.9% used multiple substances, primarily methamphetamine and opioids. The primary outcome was all-cause mortality within the first year of life, with analyses stratified by substance type and timing of exposure. Cox regression models were employed to assess mortality, with results presented as adjusted hazard ratios (aHRs) with 95% confidence intervals (CIs). A *P*-value below .05 was considered statistically significant.

**Results:**

Infant from illicit drug-exposed mothers had a higher all-cause mortality rate (0.7%) compared to the control group (0.4%). Polysubstance use, which in most cases involved methamphetamine or opioids, was significantly associated with increased mortality risk (aHR 1.53, 95% CI 1.00–2.34), whereas no single substance alone—including methamphetamine (aHR 1.38, 95% CI 0.87–2.19) or opioids (aHR 1.63, 95% CI 0.98–2.72)—showed a statistically significant association. 3,4-Methylenedioxymethamphetamine, ketamine, and cannabis were likewise not linked to increased mortality. Mortality risk increased with drug exposure during pregnancy, with borderline significant associations in the first (aHR 1.82, 95% CI 0.98–3.37) and second trimesters (aHR 1.96, 95% CI 0.99–3.86), suggesting heightened vulnerability during early to mid-gestation.

**Conclusion:**

One-year infant mortality is elevated among women with illicit substance use, with a higher proportion of deaths attributed to preterm birth and hypoxic events. The highest mortality risk was observed among those with polysubstance use. The findings underscore a dire public health issue, associating prenatal illicit substance exposure, notably multiple substances use, opioids, and methamphetamine, with heightened infant mortality rates, calling for targeted interventions and further research.

Significance Statement This study highlights a critical public health issue: the impact of prenatal illicit substance exposure on infant mortality. Analyzing data from nearly two million pregnancies over 16 years, we found that infant born to mothers with substance exposure had significantly higher all-cause mortality rates compared to unexposed controls. The risks were particularly pronounced with polysubstance use, most of which involved methamphetamine or opioids. In contrast, no single substance—including methamphetamine (aHR 1.38) or opioids (aHR 1.63)—showed a statistically significant association. These risks were heightened when exposure occurred during the first and second trimesters, and the increased mortality persisted beyond the neonatal period into the first year of life. Our findings emphasize the urgent need for interventions targeting substance use during pregnancy and for further research to understand and mitigate these risks. These results underscore the importance of integrated care models that combine maternal substance use treatment with comprehensive prenatal and pediatric health services.

## INTRODUCTION

The profound ramifications of prenatal illicit drug exposure on infant health and mortality have consistently garnered attention from the global health and scientific communities. ^1^^,^^2^ Historically, exposures to substances such as opioids and stimulants, notably cocaine, were strongly associated with heightened risks of fetal and infant mortality.^2–7^ However, despite these findings, relatively few studies have directly examined the impact of prenatal illicit drug exposure on infant mortality outcomes. Several prior studies have reported increased infant mortality rates among pregnant women with opioid use disorders.^8–12^ Yet, many of these studies have placed limited emphasis on the potential repercussions of amphetamine-type stimulants, particularly methamphetamine, which is the predominant illicit stimulant in Taiwan and several parts of East and Southeast Asia, in contrast to the cocaine-dominant patterns seen in Europe and North America. As a result, certain aspects of the risks associated with prenatal exposure to methamphetamine remain underexplored. Meanwhile, a study that took into account both opioids and methamphetamine suggested a more encompassing approach. Still, it remained limited in its reach, drawing data solely from four hospitals within a 5-year frame, without providing insights into crucial factors such as the timing of substance exposure during pregnancy or distinct ages of infant mortality.^13^ A recent review emphasized the importance of considering both opioids and methamphetamine when evaluating perinatal outcomes, noting that although these substances have different pharmacological properties, they both adversely affect embryonic development and may lead to different patterns of infant mortality depending on age. Moreover, the combined use of opioids and methamphetamine may intensify these negative effects.^14^ While such findings suggest the need for a more comprehensive approach, prior studies remain limited in scope, often restricted to select hospital-based samples without adequate consideration of the timing of illicit substance exposure or detailed cause-specific mortality. While the regional prominence of methamphetamine uses warrants attention, our study was not limited to methamphetamine; rather, we aimed to assess and compare the relative mortality risks associated with different illicit substance categories.

Evolving from these prior observations, a recurring impediment within this realm has been the prevalent dependence on self-reported data, which inevitably encounters intrinsic biases.^15^ While substantial literature has established associations between substances such as opioids, cocaine, tobacco, and alcohol with adverse perinatal outcomes,^16^^,^^17^ prior studies have also demonstrated that methamphetamine exposure is associated with increased risks of preterm birth, stillbirth, and neurodevelopmental delays.^18–20^ However, relevant data from Asian regions remain limited. In particular, the trimester-specific susceptibilities and longer-term mortality risks associated with prenatal illicit substance exposure have not been comprehensively studied in these populations. Our study aimed to address this regional knowledge gap by utilizing the Taiwan National Health Insurance Research Database to examine prenatal exposure patterns and their associations with infant outcomes. In particular, we hypothesized and investigated whether different types of illicit substance exposure—such as methamphetamine, opioids, and polysubstance use—prior to or during pregnancy are associated with increased infant mortality, and how such risks may vary by substance type and timing of exposure. By doing so, we hope to provide region-specific insights that complement existing global literature and inform targeted clinical and public health interventions.

## METHODS

### Study Participants and Data Source

We conducted this retrospective matched cohort study using the Taiwan National Health Insurance Research Database (NHIRD), maintained by the National Health Insurance program in Taiwan. Established on March 1, 1995, the program enrolled 99.9% of Taiwan’s population of 23.7 million individuals.^21^ The NHIRD interconnected with several other databases, such as the Birth Notification System (BNS), National Register of Death (NRD), Taiwan’s Maternal and Child Health Database (TMCHD), and the Integrated Illegal Drug Database (IIDD). Of particular significance, the IIDD offered information on national drug scheduling and various types of drug-related criminal records (DCR), encompassing drug consumption, possession, manufacturing, trafficking, and distribution. It stood as one among 20 illegal-drug related databases set up by various governmental agencies. Every database utilized in our study originated from Taiwan’s government administrative databases, recognized as valuable resources for population health sciences studies.^22–24^ For the cohort, data were sourced from the NHIRD, encompassing all Taiwan residents mandated to join the National Health Insurance program until either death or emigration. Individuals with health insurance coverage were included regardless of their residency status. Every health care facility in Taiwan mandatorily registered birth certificates. The Institutional Review Board of the National Health Research Institutes in Taiwan granted approval for this study (IRB number: EC1070601-E). Due to the anonymization of all patient data prior to analysis, the review board exempted the requirement for written informed consent. Our study adhered to the Reporting of studies Conducted using Observational Routinely-collected Data – Prenatal Exposures (RECORD-PE) reporting guideline.

**Figure 1:**
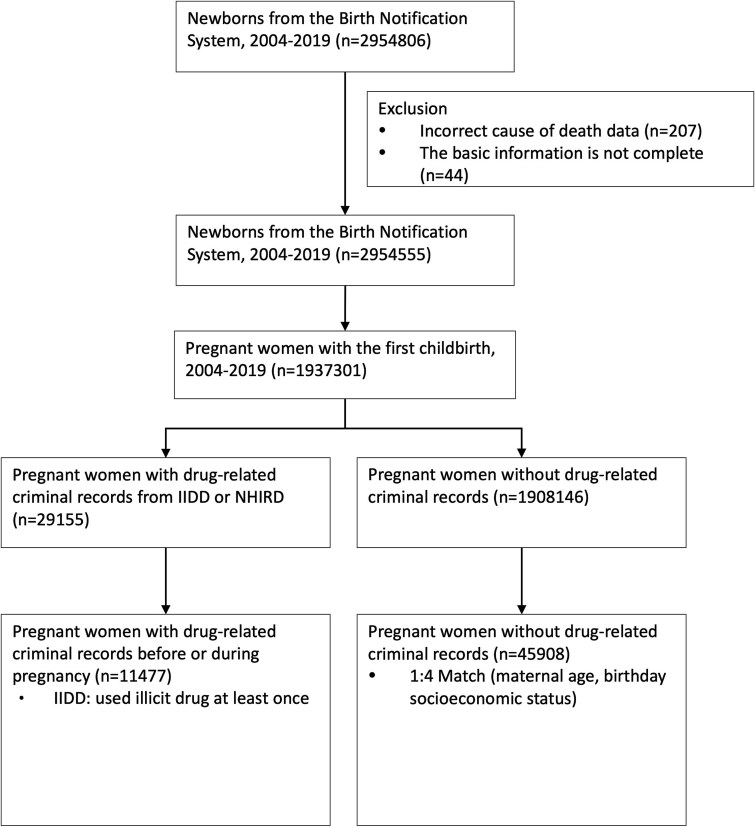
Data collection flowchart for pregnant women with illicit substance use and control group of non-users.

The focus of our research was to offer crucial evidence connecting illegal drug usage with infant mortality risks, underlining the importance of early interventions and specific prevention strategies. We scrutinized data from 1 937 301 pregnant women who had their first childbirth between 2004 and 2019, as drawn from the NHIRD and linked with the Taiwan Maternal and Child Health dataset. The IIDD was employed to identify 29 155 pregnant women with drug use disorders, of which 11 477 had at least one instance of illicit drug usage during pregnancy. A control group comprising 45 908 non-illicit drug users was assembled, matched 1:4 with illicit drug users based on parameters like maternal age, income, and year of childbirth (Figure 1). Subsequent outcome analyses were focused exclusively on all-cause mortality occurring within the first year of life.

### Prenatal Exposure to Illicit Substance

For the purpose of our study, illicit drug exposure during pregnancy was defined as any usage of opioids, methamphetamines, 3,4-Methylenedioxymethamphetamine (MDMA), cannabis, ketamine, or other similar substances. We excluded pregnant women engaged in drug possession, manufacturing, trafficking, or distribution. However, those who tested positive for drugs during urine screenings and had a drug-related criminal record documented by the police department were included. The timing of drug exposure was based on the results of urine tests conducted on the day the pregnant woman was apprehended. Therefore, the date recorded in our data is the date of apprehension, not the date of the verdict announcement. This definition provides a reference closer to the actual time of drug use, as the urine test on the day of apprehension can reflect recent drug use.

To ascertain the correlation between prenatal illicit drug exposure and infant mortality, drug exposure times were categorized into three trimesters: first, second, and third. Pregnant women with drug-related criminal records up to the end of the 12th week were considered exposed during the first trimester. Those with records from the start of the 13th week to the end of the 27th week (+6 days) were tagged under the second trimester, while records from the start of the 28th week indicated exposure during the third trimester. This classification enabled us to assess the potential influence of drug exposure timing on infant death.^24^

### Outcome Measures

The primary outcome measure for our cohorts was all-cause mortality. Our study, built upon the NRD, centered on death as the principal outcome. We observed from the date of birth until either the date of death, the end of the study period (December 31, 2019), or the date when the insurance was canceled without a re-enrollment record. With the study period extending up to 16 years, we could investigate trends over an extended duration. We aimed to ascertain whether drug use during or prior to pregnancy contributed to mortality within the first year of life, as deaths in infancy are more plausibly attributable to prenatal exposures and early-life health conditions.

In addition to the primary outcome, we examined several secondary outcomes to further characterize the associations between maternal illicit substance use and infant mortality. These included analyses stratified by type of substance exposure (methamphetamine, opioids, polysubstance use) and by timing of exposure (pre-pregnancy and each trimester). Multivariable models were used to identify specific substances and critical exposure periods associated with increased risk of infant mortality.

### Confounders

We identified and included a set of confounders in our analysis based on established literature and clinical relevance to infant mortality outcomes among drug-exposed pregnancies. We derived information related to confounding factors from the NHIRD and incorporated into all adjusted models to control for potential bias. Maternal characteristics included age, socioeconomic status (categorized into two tiers based on the payroll bracket table disseminated by the National Health Insurance Administration of the Ministry of Health and Welfare) expressed in New Taiwan Dollars (TWD),^25^ and comorbidities such as diabetes mellitus,^26^ hypertension, psychiatric disease, human immunodeficiency virus, viral hepatitis, tuberculosis,^27^ obesity, and alcohol use disorders. Comorbidities were identified using ICD-9-CM or ICD-10-CM codes, requiring at least three outpatient visits or one inpatient admission prior to pregnancy. Pregnancy-related complications (e.g. anemia, preeclampsia, and gestational diabetes mellitus^28^) and neonatal conditions (e.g. congenital malformations and hypoxia) were identified through inpatient or outpatient claims.^25^^,^^28^ Additionally, we controlled for birth-related factors such as gestational age (^29^<37 vs. ≥37 weeks), birth weight^30^ (<2500 g vs. ≥2500 g), and neonatal sex sourced from the BNS. These confounders were included in all multivariable models to adjust for potential confounding factors and to accurately estimate the association between prenatal illicit substance exposure and infant mortality during the first year of life.

### Statistical Analysis

For statistical analysis, categorical variables were presented as percentages and compared using chi-square tests. We utilized Cox proportional hazards regression models to estimate adjusted hazard ratios (aHRs) and 95% confidence intervals (CIs) for the association between prenatal illicit drug exposure and all-cause mortality among infant. Models were adjusted for the full set of confounders listed above. A *P*-value <.05 was considered statistically significant. All analyses were conducted using SAS version 9.4 (SAS Institute, Cary, NC, USA).

## RESULTS

Table 1 delineates the demographic characteristics of the study cohort, encompassing 11 477 pregnant women exposed to illicit drugs and a control group of 45 908 unexposed women. Infant from drug-exposed mothers had an overall all-cause mortality rate of 1.0%, double the 0.5% observed in the control group. Specifically, infant mortality within the first year of life was 0.7% in the exposed group versus 0.4% in the control group, which became the primary focus of subsequent analyses. Moreover, neonatally, these infant exhibited higher incidences of hypoxia, low birth weight (<2500 g), and preterm births (<37 weeks) compared to those born to unexposed mothers. Maternally, all comorbidities, except obesity, were more prevalent among drug-exposed women.

**Table 1:** Demographic and Clinical Characteristics of Pregnant Women Exposed to Illicit Substances and Unexposed Control Group.

**Characteristic**	**Matched unexposed group**	**Drug exposed group**	**Total number**	***P*-value**
	**(n = 45 908)**	**(n = 11 477)**		
	**N (%)**	**N (%)**		
**Maternal age**				>.999
** <25**	14 000 (30.5)	3500 (30.5)	17 500	
** 25-29**	15 192 (33.1)	3798 (33.1)	18 990	
** 30-34**	11 092 (24.2)	2773 (24.2)	13 865	
** ≥35**	5624 (12.3)	1406 (12.3)	7030	
**Maternal comorbidity**				
** Diabetes mellitus**	765 (1.7)	252 (2.2)	1017	<.001
** Hypertension**	392 (0.9)	121 (1.1)	513	.041
** Psychiatric disease**	1103 (2.4)	1192 (10.4)	2295	<.001
** Viral hepatitis**	737 (1.6)	422 (3.7)	1159	<.001
** Tuberculosis**	205 (0.4)	83 (0.7)	288	<.001
** Obesity**	231 (0.5)	41 (0.4)	272	.042
** Alcohol use disorder**	57 (0.1)	97 (0.8)	154	
**Pregnancy-related complication**				
** Anemia**	1421 (3.1)	353 (3.1)	1774	.914
** Preeclampsia**	580 (1.3)	145 (1.3)	725	>.999
** Gestational diabetes mellitus**	832 (1.8)	114 (1.0)	946	<.001
**Neonatal gender**				>.999
** Female**	22 008 (47.9)	5502 (47.9)	27 510	
** Male**	23 900 (52.1)	5975 (52.1)	29 875	
**Number of babies**				.041
** Singleton**	45 232 (98.5)	11 337 (98.8)	56 569	
** Multiple**	676 (1.5)	140 (1.2)	816	
**Gestational age**				<.001
** ≥37 weeks**	42 163 (91.8)	9991 (87.1)	52 154	
** <37 weeks**	3745 (8.2)	1486 (12.9)	5231	
**Birthweight**				<.001
** ≥2500 g**	42 057 (91.6)	9845 (85.8)	51 902	
** <2500 g**	3851 (8.4)	1632 (14.2)	5483	
**Neonatal comorbidity**				
** Congenital malformation**	2794 (6.1)	693 (6.0)	3487	.848
** Hypoxia**	313 (0.7)	130 (1.1)	443	<.001
**Infant’s Death**	239 (0.5)	111 (1.0)	350	<.001
** Before 1 year old**	170 (0.4)	75 (0.7)	245	<.001
** After 1 year old**	69 (0.2)	36 (0.3)	105	<.001

Table 2 presents the multivariate analysis of factors associated with mortality within the first year of life. After adjusting for confounders, illicit substance exposure during pregnancy was associated with an increased risk of infant mortality (HR 1.31, 95% CI 0.99–1.73), although the association did not reach statistical significance.

**Table 2:** Multivariable Analysis of Factors Associated with Infant Mortality Risk in Prenatal Illicit Drug Exposure.

**Variables**	**HR**	**95% CI**	
**Illicit drugs exposure**			
** No**	1.00		
** Yes**	1.31	0.99	1.73
**Maternal age**			
** <25**	1.00		
** 25-29**	0.78	0.57	1.08
** 30-34**	**0.58**	**0.40**	**0.84**
** ≥35**	1.08	0.75	1.55
**Maternal comorbidity**			
** Diabetes mellitus**	**1.97**	**1.06**	**3.66**
** Hypertension**	1.76	0.83	3.72
** Psychiatric disease**	1.28	0.77	2.12
** Viral hepatitis**	0.59	0.22	1.59
** Tuberculosis**	^a^		
** Obesity**	^a^		
** Alcohol use disorder**	^a^		
**Pregnancy-related complication**			
** Anemia**	1.18	0.64	2.17
** Pre-eclampsia**	0.80	0.39	1.67
** Gestational diabetes mellitus**	1.70	0.80	3.64
**Neonatal gender**			
** Female**	1.00		
** Male**	0.99	0.77	1.27
**Number of babies**			
** Singleton**	1.00		
** Multiple**	0.70	0.38	1.27
**Gestational age**			
** ≥37 weeks**	1.00		
** <37 weeks**	4.16	2.94	5.88
**Birthweight**			
** ≥2500 g**	1.00		
** <2500 g**	**3.74**	**2.65**	**5.29**
**Neonatal comorbidity**			
** Congenital malformation**	1.16	0.78	1.74
** Hypoxia**	**2.39**	**1.50**	**3.84**

Bold values indicate statistical significance (95% CI does not include 1). Abbreviations: HR, hazard ratio; CI, confidence interval.

a The number of cases was too small to disclose owing to concerns about the privacy of identifying patients.

**Figure 2:**
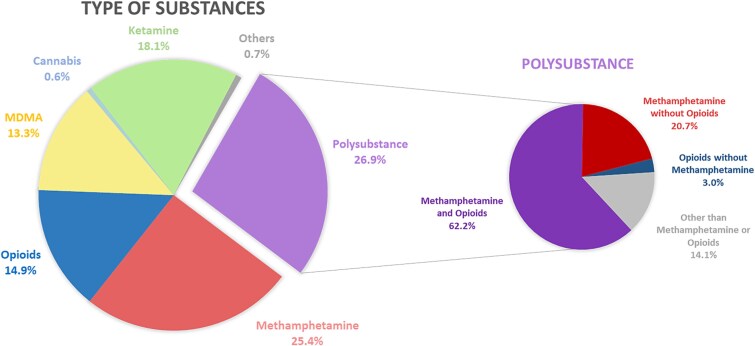
Types of illicit substance exposure among pregnant women. Abbreviation: MDMA, 3,4-Methylenedioxymethamphetamine. In the left figure, any illicit drug usage that accounted for ˂0.5% was categorized under “others”. In the right figure, the categorization of “polysubstance” is exemplified as follows: If pregnant women use methamphetamine and ketamine but do not use opioids, they are categorized under the “Methamphetamine without opioids” subgroup; if pregnant women use cannabis and MDMA but do not use opioids or methamphetamine, they are categorized under the “other than methamphetamine or opioids” subgroup.

Figure 2 outlines the categories of illicit substances used by the 11 477 mothers who were exposed. The majority used multiple substances (polysubstance) at 26.9%. For those who used a single substance, methamphetamine was the most common at 25.4%, followed by ketamine (18.1%), opioids (14.9%), MDMA (13.3%), and cannabis (0.6%). Any illicit drug usage accounting for ˂0.5% was grouped under “others”. Among multiple substances users, 82.9% included methamphetamine in their mix, 65.2% included opioids, and 85.9% incorporated either methamphetamine or opioids.

Table 3 delves into the relationship between prenatal illicit drug exposure and infant mortality risk. All-cause mortality rates following prenatal exposure were: polysubstance (0.8%), opioids (1.0%), and methamphetamine (0.7%). After adjustment for potential confounders, elevated risks were observed across all exposure groups. Polysubstance use, which in most cases involved methamphetamine or opioids, was significantly associated with increased mortality risk (aHR 1.53, 95% CI 1.00–2.34), whereas no single substance alone—including methamphetamine (aHR 1.38, 95% CI 0.87–2.19) or opioids (aHR 1.63, 95% CI 0.98–2.72)—showed a statistically significant association. MDMA, ketamine, and cannabis were likewise not linked to increased mortality. Mortality risk increased with drug exposure during pregnancy, with borderline significant associations in the first (aHR 1.82, 95% CI 0.98–3.37) and second trimesters (aHR 1.96, 95% CI 0.99–3.86), suggesting heightened vulnerability during early to mid-gestation.

**Table 3:** Multifactorial Analysis of Infant Mortality Risk Associated with Types of Prenatal Illicit Drug Exposure.

**Illicit drugs exposure**	**Mortality%** **(n/Total)**	**HR**	**95% CI**	
**No**	0.4 (170/45908) 0.8 (26/3092) 0.7 (21/2915) 1.0 (17/1713) 0.3 (4/1523) 0(0/69) 0.3 (6/2082)			
**Polysubstances**		**1.53**	**1.00**	**2.34**
**Methamphetamine**		1.38	0.87	2.19
**Opioids**		1.63	0.98	2.72
**MDMA**		0.76	0.28	2.06
**Cannabis**		–		
**Ketamine**		0.71	0.31	1.61
**Others**	^a^	2.97	0.41	21.37

Bold values indicate statistical significance (95% CI does not include 1). Abbreviations: HR, hazard ratio; CI, confidence interval; MDMA, 3,4-Methylenedioxymethamphetamine.

a The number of cases was too small to disclose owing to concerns about the privacy of identifying patients.

Table 4 examines the association between timing of prenatal illicit substance exposure and infant mortality risk within the first year of life, based on multivariable analyses adjusted for confounders. Exposure during the first trimester was associated with an aHR of 1.82 (95% CI 0.98–3.37), and exposure during the second trimester yielded an aHR of 1.98 (95% CI 0.99–3.86), both reflecting borderline statistical significance. Pre-pregnancy exposure showed no statistically significant association (aHR 1.16, 95% CI 0.84–1.60).

**Table 4:** Multifactorial Analysis of Infant Mortality Risk Associated with the Timing of Prenatal Illicit Drug Exposure.

**Illicit drugs exposure**	**Mortality% (n/Total)**	**HR**	**95% CI**	
**No**	0.4 (170/45908) 0.5 (52/9770) 1.2 (11/893) 1.6 (9/579) 1.3 (3/235)			
**Before Pregnancy**		1.16	0.84	1.60
**First trimester**		1.82	0.98	3.37
**Second trimester**		1.96	0.99	3.86
**Third trimester**		1.63	0.52	5.13

Abbreviations: HR, hazard ratio; CI, confidence interval.

## DISCUSSION

Our study highlighted the association between prenatal exposure to illicit substances and increased risk of infant mortality within the first year of life, underscoring the importance of addressing substance use during pregnancy to improve neonatal and infant outcomes. Among the various substances examined, polysubstance use emerged as the most concerning pattern, reflecting the compounded risk of multiple toxic exposures during fetal development. The fact that most polysubstance cases involved methamphetamine or opioids may explain the elevated risk observed, even when individual substances did not reach statistical significance. This finding suggests a potential synergistic effect of combined substance exposure, or that polysubstance use may serve as a marker of more severe or chaotic patterns of substance use and associated social risk factors. Additionally, the trend toward higher mortality risk with first- and second-trimester exposures highlights the critical importance of early prenatal screening and intervention. These results align with the known vulnerability of organogenesis and early fetal development to teratogenic insults, reinforcing the need for timely and sustained maternal care throughout pregnancy.

Polysubstances use, especially during pregnancy, was a salient feature of our findings, with a significant 26.9% of prenatal illicit drug exposures attributed to multiple substances use—a figure that resonates with real-world clinical scenarios. Our data underscored significant mortality risks associated with prenatal multiple substances exposure, particularly emphasizing methamphetamine and opioids. In our cohort, an overwhelming 82.9% of multiple substances exposures involved methamphetamine, while opioids featured in 65.2% of cases. When examined independently, the link between these substances and increased all-cause mortality was stark. Despite their different pharmacological properties, both substances adversely impact embryonic development,^3^ which might lead to diverse causes of infant mortality depending on age. Models of cumulative risk suggest that prenatal exposure to multiple substances contributes to a continuum of impairment in perinatal outcomes, with greater risks observed compared to exposure to a single substance. This synergistic effect may explain the heightened mortality risks we observed among infant of mothers with polysubstance use during pregnancy.^14^^,^^31^

Consistent with previous research, our study drew attention to the elevated infant mortality rate due to illicit substance exposure during pregnancy. For instance, a Tennessee study reported increased infant mortality associated with opioid use disorders over an 11-year period.^8^ Another study from Iran identified heightened neonatal mortality risks in substance-using pregnant women, including those using opioids and amphetamines.^13^ Extending beyond the limitations of prior hospital-based or regional studies, our nationwide, population-based design allowed for a more granular assessment of exposure timing across gestation. The observed pattern of higher mortality risk with exposure during the first and second trimesters, even if not statistically definitive, is biologically plausible and highlights early gestation as a particularly sensitive window. This finding supports the hypothesis that prenatal drug exposure may disrupt critical phases of fetal development, such as organogenesis and central nervous system maturation. It also emphasizes the importance of early detection and intervention strategies during pregnancy to mitigate adverse outcomes.^32^^,^^33^

Illicit drug use is often accompanied by psychosocial risk factors such as poverty, maternal mental health disorders, and lack of social support, which may persist into the postpartum period and threaten infant development and survival. While these complex postnatal factors may contribute to long-term child outcomes, our study focused exclusively on infant mortality within the first year of life, which is more likely to reflect biological mechanisms directly related to prenatal exposures. Further research incorporating psychosocial and longitudinal data will be necessary to fully explore long-term developmental consequences.

Our study has several limitations. First, although infant mortality was higher in the exposed group, the number of deaths remained relatively small, limiting the power to explore specific causes in detail. As a result, we focused on all-cause mortality as the primary outcome. To offer preliminary insights, we conducted an exploratory classification of deaths into accidental and non-accidental categories. Accidental deaths included unintentional injuries and sudden infant death syndrome (SIDS), pointing to the potential influence of environmental and caregiving factors. Non-accidental deaths encompassed congenital anomalies, chromosomal disorders, perinatal conditions, malignancies, neurological disorders such as cerebral palsy, and infections. Notably, among the non-accidental deaths, congenital anomalies appeared frequently, but the data did not allow us to distinguish between major and minor anomalies—another constraint of this study. Despite these limitations, the preliminary categorization provides a useful foundation for future research into specific mechanisms linking prenatal substance exposure and infant mortality. Second, our database lacks key postnatal risk factors, including maternal relapse, caregiving quality, access to resources, nutrition, housing conditions, child placement, and healthcare accessibility. These may confound the risk of postnatal death, especially the long-term impact beyond infancy. Interpretations should therefore be made with caution. Third, critical perinatal clinical information was unavailable, including adequacy of prenatal care, maternal infections (e.g. tuberculosis, group B Streptococcus), severity of prematurity, neonatal intensive care needs, hospitalization duration, and discharge planning—all of which can influence infant mortality. Moreover, some observed correlates of mortality (e.g. hypoxia) may reflect underlying causes such as birth asphyxia or chronic health conditions, but the exact mechanisms could not be determined. Fourth, the identification of substance exposure relied on the Illicit Drug Database (IIDD), which is based primarily on police reports and urine test records, potentially missing unreported cases and underestimating actual exposure prevalence. Fifth, our study could not capture certain behavioral exposures such as smoking or subclinical alcohol use. Although we adjusted for diagnosed alcohol use disorder, broader, undiagnosed alcohol exposure was not included in the analysis. Past studies underscore the detrimental impact of both tobacco and alcohol on neurodevelopment during prenatal opioid use, with confirmed adverse effects on infant mortality rates.^34^ Sixth, while gestational age, birthweight, and neonatal comorbidities were all retained in the model due to their independent clinical relevance, we did not assess collinearity or include interaction terms, which may warrant further investigation in future studies. Lastly, given that our sample was derived from Taiwan, with its unique patterns of substance exposure and healthcare system, the generalizability of our findings to other regions remains limited. For example, our cohort had a notable underrepresentation of women exposed to cannabis during pregnancy, making up only 0.6% of all drug exposures. Remarkably, there were no recorded infant deaths in this group throughout the study. This observation starkly contrasts with a previous U.S. study which showed increased infant mortality risks within the first year after prenatal cannabis exposure.^35^ The difference in these findings beckons more in-depth exploration in subsequent research. Future studies should validate these results in different national contexts.

Despite these limitations, our study methodologically possesses several key strengths. The population-based cohort design effectively reduced selection bias, while the study’s prospective approach minimized the risk of reverse causality. We performed comprehensive adjustments to account for multiple potential confounders. One of the main pillars of our research was the utilization of a vast nationwide database, encompassing 57 385 mother–child pairs over a span of 16 years. This extensive dataset, in conjunction with Taiwan’s universal single-payer healthcare system and tools such as the IIDD, BNS, National Register of Death, and Taiwan’s Maternal and Child Health Database, allowed for the meticulous identification of mother–child pairs and detailed tracking of their prenatal illicit drug exposures. This rigorous structure considerably reduced both selection and misclassification biases, solidifying the validity of our results. The vast scope of our study assured sufficient statistical power. Regarding the charges recorded in the database, our study focuses on pregnant women with substance use disorders in Taiwan. According to local regulations, when these women enter the judicial process and are either pregnant or within two months postpartum, the execution of imprisonment or detention is suspended, or they are denied entry into correctional facilities. This policy is designed to ensure that these women receive comprehensive prenatal and postnatal healthcare services, prevent environments and behaviors that could perpetuate substance use, and protect the health of both mother and child from ongoing drug-related risks. Therefore, under this regulatory framework, such cases do not involve incarceration that would result in situations where infant are not under maternal care.

In conclusion, our study suggests an elevated risk of infant mortality within the first year of life associated with prenatal illicit drug exposure, particularly among women with polysubstance use. Although statistical significance was not consistently achieved, the observed trends highlight potential clinically relevant risks that warrant attention. These findings underscore the need for targeted clinical interventions, early screening, and public health policies in Taiwan to address maternal substance use and its potential impact on infant survival. Further research is necessary to confirm these associations and elucidate underlying mechanisms.

## Supplementary Material

Supplementary_R2_pyaf046

## Data Availability

The data for this study are not publicly available. To protect privacy, the Taiwanese government only allows researchers to analyze data from the Health and Welfare Data Science Center in selected computer rooms of the center. No individual data can be brought outside the center, and researchers can only bring away aggregate statistical results based on raw data. To use the data, a researcher has to apply for permission of using data within the center. To apply for such permission, a researcher has to submit an IRB approval concerning the data use and has to pay for using data.
