# Supplementary material for: Illicit substance exposure in pregnancy and infant mortality risk: a nationwide Taiwan study
Source: Int J Neuropsychopharmacol. 2025 Jul 9;28(8):pyaf046. doi: 10.1093/ijnp/pyaf046 (PMC12343109; doi:10.1093/ijnp/pyaf046)
Supplement: Supplementary_R2_pyaf046 [file supplementary_r2_pyaf046.docx]

**Supplementary Table. Cause-specific infant mortality within the first year of life among drug-exposed and unexposed groups.** This table presents the distribution of cause of death, based on ICD-9-CM and ICD-10-CM codes, comparing infants born to mothers with illicit substance exposure versus matched unexposed controls. Each cause of death is categorized according to major diagnostic groups (e.g., congenital anomalies, perinatal conditions, accidental injuries, SIDS, pneumonia). For each group, the number of deaths (n) and corresponding percentage (%) are shown. P-values are derived from chi-square or Fisher’s exact tests comparing proportions between exposed and unexposed groups. Notably, deaths due to accidental injuries, sudden infant death syndrome (SIDS), pneumonia, and unspecified causes (e.g., R99) were significantly more common among the substance-exposed group. These findings underscore the increased risk of preventable and modifiable causes of mortality in infants prenatally exposed to illicit substances.

| **Cause of death** | **ICD-9-CM** | **ICD-10-CM** | **Drug exposed group**  **n (%)** | **Matched unexposed group**  **n (%)** | **P-value** |
| --- | --- | --- | --- | --- | --- |
| Congenital malformations, deformations, and chromosomal abnormalities | 740-759 | Q00-Q99 | 16 (0.12) | 47 (0.09) | 0.284 |
| Conditions originating in the perinatal period | 760-779 | P00-P99 | 23 (0.18) | 74 (0.14) | 0.361 |
| Accidental injuries | 800-949 | V00-V99, W00-W99, X00-X59 | 23 (0.18) | 33 (0.06) | <0.001 |
| Malignant neoplasms or infantile cerebral palsy | 140-208, 343 | C00-C97, G80 | 3 (0.02) | 15 (0.03) | 0.724 |
| Sudden infant death syndrome (SIDS) | 7980 | R95 | 14 (0.11) | 15 (0.03) | <0.001 |
| Pneumonia | 480-486 | J12-J18 | 5 (0.04) | 6 (0.01) | 0.035 |
| Unknown or unspecified | 798.1, 798.2, 799.9 | R99 | 8 (0.06) | 5 (0.01) | <0.001 |
